# Telerehabilitation Readiness, Knowledge, and Acceptance of Future Physiatrists in the Philippines: An Online Survey During the COVID-19 Pandemic

**DOI:** 10.3389/fresc.2022.921013

**Published:** 2022-06-03

**Authors:** Carl Froilan D. Leochico, Marc Francis J. Perez, Jose Alvin P. Mojica, Sharon D. Ignacio

**Affiliations:** ^1^Department of Rehabilitation Medicine, Philippine General Hospital, University of the Philippines Manila, Manila, Philippines; ^2^Department of Physical Medicine and Rehabilitation, St. Luke's Medical Center, Global City, Philippines

**Keywords:** telemedicine, telerehabilitation, remote rehabilitation, virtual rehabilitation, residency training, education, COVID-19, healthcare delivery

## Abstract

**Background:**

Clinical, educational, and research interest in telerehabilitation has not been widely explored until the COVID-19 pandemic. Amid the enduring pandemic, telerehabilitation remains part of the daily service, academic, and research responsibilities of residents in various training institutions worldwide.

**Objective:**

To determine the Rehabilitation Medicine residents' current levels of telerehabilitation readiness, knowledge, and acceptance, their pattern of beliefs about telerehabilitation, and the factors affecting their readiness.

**Methods:**

All bona fide residents from all training institutions in the Philippines were invited to participate in an online survey evaluating the following constructs: technological readiness (using the Technological Readiness Index or TRI 2.0); telerehabilitation knowledge (using an original multiple-choice examination); and telerehabilitation acceptance (using the Unified Theory of Acceptance and Use of Technology questionnaire). A pre-test and pilot test were conducted. The TRI responses were classified according to technology adoption segments to determine the respondents' pattern of beliefs about telerehabilitation.

**Results:**

Sixty-two residents participated (86.1% response rate). They had good telerehabilitation readiness (3.3 ± 0.4 out of 5), fair telerehabilitation knowledge (2.1 ± 1.1 out of 5), and excellent telerehabilitation acceptance (4.5 ± 0.6 out of 5). The majority were classified either as telerehabilitation skeptics (38.7%), pioneers (19.4%), or explorers (19.4%). The factors that significantly influenced telerehabilitation readiness were optimism, innovativeness, discomfort, and insecurity (*p* < 0.05).

**Conclusion:**

Despite having favorable levels of telerehabilitation readiness and acceptance, the Rehabilitation Medicine residents showed fair telerehabilitation knowledge. Our results suggest the need for formal education and training on virtual rehabilitation care during residency.

## Introduction

Telerehabilitation, a branch of telemedicine, is an emerging method of delivering rehabilitation services through information and communications technology to connect patients and clinicians and minimize the barriers of distance, time, and cost (1). Specific telerehabilitation assessments and interventions using various computer- or gadget-based applications vary based on the patients' rehabilitation-related needs and resources and may include teleconsultations with specialists, teletherapy services (e.g., virtually facilitated exercise program, home instructions), and/or remote physiologic monitoring using body sensors technology (2).

With the rise of new technologies and “overcoming of the initial skepticism to which every new technology is subjected” (3), there has been an increase in the number of patients treated via telerehabilitation across the continuum of rehabilitation care and for various disabling conditions even before the pandemic (4). Catalyzed by the COVID-19 crisis, telerehabilitation is now being explored more widely to circumvent the persistent lack of in-person patient service in clinical, academic (undergraduate and postgraduate), and research settings especially in developing countries like the Philippines, which went through one of the longest lockdowns in the world (5–7). In the western region, a large descriptive retrospective study consisting of a sample of 222, 680 patients in the United States has considered synchronous video- or audio-based telerehabilitation as an alternative care model for different orthopedic and non-orthopedic cases during the pandemic (8). To this date, however, there is no internationally agreed telerehabilitation guideline possibly since different parts of the world may have various contexts, needs, and resources. In some countries in Southeast Asia, for instance, telerehabilitation guidelines were found to varying degrees of breadth and depth (9). Therefore, planning a telerehabilitation initiative may entail considerations of a multitude of human, organizational, and technical factors applicable to their respective setting (9, 10).

One way to prepare for a telerehabilitation program is to understand the readiness of potential target-users (i.e., patients, families, clinicians) for the technology (11). The uptake of telerehabilitation lies not solely in the hands of current Rehabilitation Medicine specialists, but those of the next generations as well. The current and future residents training for the specialty will eventually be the key persons or drivers of rehabilitation technologies. The residents who have been on the frontline during this pandemic are a rich source of first-hand experiences in the COVID-19 battlefield and may have a lot of ideas as to how rehabilitation care could be better delivered amid and beyond the pandemic. To our knowledge, the potential factors influencing this group of stakeholders' intentions to use telerehabilitation have not been explored. Evaluating and addressing the determinants of telerehabilitation readiness among different stakeholders can help administrators to develop more applicable and user-friendly telehealth-related programs, considering the former's technological capacity, knowledge, acceptance, preferences, and needs (2, 12).

Hence, the present study aimed to determine the Rehabilitation Medicine residents' baseline levels of telerehabilitation readiness, knowledge, and acceptance, their pattern of beliefs about telerehabilitation, and the factors affecting their readiness for telerehabilitation. The results of our study may serve as basis for program development or evaluation and capacity-building interventions related to improving telerehabilitation-related teaching and learning programs, research, and service delivery.

This study is founded on well-established concepts embedded in the Technology Readiness Index (TRI) version 2.0 questionnaire (13) and the Unified Theory of Acceptance and Use of Technology (UTAUT) model (14), wherein the outcome of interest is the actual intent to use a certain technology, which in this study is telerehabilitation. We define telerehabilitation readiness in this study as a resident's propensity to adopt and embrace this emerging technology for service, training, and research (6, 13). Based on the TRI questionnaire, technological readiness is affected positively by “motivators” (i.e., optimism and innovativeness) and negatively by “inhibitors” (i.e., discomfort and insecurity) (13). Meanwhile, telerehabilitation acceptance is the act of receiving and agreeing with the idea of using the technology to provide rehabilitation services over a distance (11). Adapted from the UTAUT model, the following factors may affect telerehabilitation acceptance: performance expectancy, effort expectancy, attitude, social influence, facilitating conditions, self-efficacy, anxiety, and behavioral intention (14, 15). Lastly, telerehabilitation knowledge pertains to the extent of information acquired by an individual through personal or vicarious experiences and any form of education or training related to the theoretical and practical understanding of the technology and its process.

## Methods

This was a cross-sectional study approved by the institutional research board and the Philippine Board of Rehabilitation Medicine (PBRM). We employed total enumeration of all bona fide residents across all year levels (i.e., 1^st^-3^rd^ year and chief residents) training in all of the six Rehabilitation Medicine residency programs in the Philippines during the period of data collection (September to November 2020). Based on the census of residents (i.e., total number: 72) recognized by the PBRM, the sample size was computed at 62 with 0.05 margin of error. The eligibility criteria included access to stable Wi-Fi broadband and ability to provide full voluntary informed consent.

The entire survey could be accomplished within 10–20 min on Google Form™. The initial part collected the following demographic data: age, sex, residency training institution, year level in training, and prior telemedicine/ telerehabilitation experience. The survey consisted of three questionnaires, namely: (1) the 16-item TRI version 2.0 to evaluate technology readiness; (2) an original 5-item multiple-choice test to evaluate telerehabilitation knowledge; and (3) the 31-item UTAUT questionnaire to evaluate telerehabilitation acceptance.

The validity and reliability of the survey were established through a pre-test. In addition, a pilot test involving 12 residents randomly selected from different training institutions was conducted prior to data collection to obtain feedback on how the wording of the survey and study implementation could be improved. During the actual data collection, respondents were given up to 3 months to complete the survey at their most convenient time. All data gathered remained anonymous and confidential. Descriptive (e.g., measures of central tendency, frequencies, and percentages) and inferential statistics (e.g., linear regression) were used to analyze the results with 95% confidence interval.

### Telerehabilitation Readiness

The modified version of the TRI, also known as the Abbreviated Technology Readiness Index by Parasuraman and Colby, is a valid and reliable tool (Cronbach's alpha = 0.81) that measures overall technological readiness (13). Permission to use the tool was secured from the TRI developers (Rockbridge Associates, Inc.). Reliability testing of the questionnaire that we modified for our intended population was conducted through a pre-test of 12 randomly selected residents training in the study institution. The 16-item questionnaire, which evaluated the “motivators” and “inhibitors” affecting one's technological readiness, was answerable using a 6-point Likert scale per item as follows: strongly agree = 6; somewhat agree = 5; neutral = 4; somewhat disagree = 3; strongly disagree = 2; or not sure = 1. Each item corresponded to one of four technology readiness (TR) dimensions grouped as follows: (1) positive themes: optimism and innovativeness; and (2) negative themes: insecurity and discomfort. There were 4 items that examined each dimension, and an average score for each dimension was computed per respondent. The total TR score was computed per respondent using the following formula: TR = (Optimism + Innovativeness + [6-Insecurity] + [6-Discomfort]) / 4 (13, 15). The total scores were directly proportional to technological readiness and interpreted as follows: 1.00−1.80 = poor, 1.81−2.60 = fair, 2.61−3.40 = good, 3.41−4.20 = very good, 4.21−5.00 = excellent telerehabilitation readiness. Based on the anonymized responses, the TRI developers grouped each respondent into one of the following categories in the order of decreasing likelihood of technology adoption: explorers, pioneers, skeptics, hesitators, and avoiders (13, 15).

### Telerehabilitation Knowledge

Three professors in the study institution, who are considered early adopters of the technology and have delivered local and international talks on telerehabilitation, convened to come up with 5 original multiple-choice questions to evaluate the residents' basic telerehabilitation knowledge. They considered the questions as must-knows in providing a quality telerehabilitation service. The questions were subjected to a pre-test and modified accordingly to achieve an acceptable Cronbach's alpha of at least 0.70. To ensure understandability of the questions, a pilot test was also done. Each correct answer was given 1 point, and the sum of scores was computed for each respondent and interpreted as follows: 1.00−1.80 = poor, 1.81−2.60 = fair, 2.61−3.40 = good, 3.41−4.20 = very good, 4.21−5.00 = excellent telerehabilitation knowledge.

### Telerehabilitation Acceptance

Telerehabilitation acceptance was evaluated using the validated 31-item UTAUT questionnaire, which consists of the following constructs: performance expectancy (PE), effort expectancy (EE), social influence (SI), facilitating conditions (FC), attitude (AT), anxiety (AX), self-efficacy (SE), and behavioral intention (BI) to use the technology (16–18). Each item consisted of Likert scale responses as follows: strongly agree = 6; somewhat agree = 5; neutral = 4; somewhat disagree = 3; strongly disagree = 2; and not sure = 1. Reliability testing of the questionnaire to suit the present study's target population was conducted following the same method previously described for telerehabilitation readiness. Three or more items evaluated one particular construct. The means and standard deviations were used to summarize the data per item and per construct. The overall mean of the constructs per respondent was interpreted as follows: 1.00−1.80 = poor, 1.81−2.60 = fair, 2.61−3.40 = good, 3.41−4.20 = very good, 4.21−5.00 = excellent telerehabilitation acceptance.

Lastly, to determine the factors that influenced the respondents' readiness for telerehabilitation, the mean TRI scores were used. Since data indicating telerehabilitation readiness were in ratio-continuous form, linear regression was done. Tests of assumption (e.g., linearity, normality, heteroscedasticity, multicollinearity) were performed beforehand to ensure that the regression model test statistics were applicable. Wherever data were not suitable for such tests, non-parametric test statistics were conducted.

## Results

A total of 62 out of 72 residents participated (86.1% response rate). The demographic characteristics of the respondents are presented in Table 1. Of special note, more than 50% had telerehabilitation experience prior to the survey.

**Table 1:** Demographic profile of the respondents (*N* = 62).

**Characteristics**	***n* (%) or Mean ±SD**
**Sex**	
Female	33 (53.2)
Male	29 (46.8)
Age, years	30.3 ± 2.7
**Residency training institution**	
Philippine General Hospital	20 (32.3)
Philippine Orthopedic Center	17 (27.4)
University of Santo Tomas	9 (14.5)
Ospital ng Makati	7 (11.3)
Veterans Memorial Medical Center	5 (8.1)
St. Luke's Medical Center	4 (6.5)
**Year level**	
1st	23 (37.1)
2nd	18 (29.0)
3rd	17 (27.4)
Chief residency (if extra year)	4 (6.5)
With prior telerehabilitation experience	33 (53.2)

The overall TRI score of the respondents was 3.3 ± 0.4 out of 5, interpreted as good telerehabilitation readiness. The TR dimension with the highest mean score was optimism, while the one with the lowest score was discomfort (Table 2). Based on the TRI responses, the majority were classified as telerehabilitation skeptics (*n* = 24, 38.7%), followed by pioneers (*n* = 12, 19.4%), explorers (*n* = 12, 19.4%), hesitators (*n* = 11, 17.7%), and avoiders (*n* = 3, 4.8%).

**Table 2:** Telerehabilitation readiness of the respondents (*N* = 62) based on the modified Technological Readiness Index (TRI) version 2.0^*^.

**Items per TRI dimension**	**Mean ±SD^**†**^**
**Optimism**	
Telerehabilitation can contribute to a better quality of life.	5.4 ± 0.6
Telerehabilitation can provide users with more freedom of mobility.	5.1 ± 0.8
Telerehabilitation can give users more control over their daily lives.	4.7 ± 1.0
Telerehabilitation can make me more productive in my personal life.	4.7 ± 0.8
Average score for optimism	**5.0** **±0.6**
**Innovativeness**	
Other people come to me for advice on new technologies.	4.5 ± 1.2
In general, I am among the first in my circle of friends to acquire new technology when it appears.	3.6 ± 1.1
I can usually figure out new high-tech products and services without help from others.	4.4 ± 1.1
I keep up with the latest technological developments in my areas of interest.	4.6 ± 1.1
Average score for innovativeness	**4.3** **±0.8**
**Discomfort**	
When I get technical support from a provider of a high-tech product or service, I sometimes feel as if I am being taken advantage of by someone who knows more than I do.	3.3 ± 1.1
Technical support lines are not helpful because they don't explain things in terms I understand.	3.4 ± 0.8
Sometimes, I think that technology systems are not designed for use by ordinary people.	3.9 ± 1.1
There is no such thing as a manual for a high-tech product or service that's written in plain language.	3.6 ± 1.1
Average score for discomfort	**3.6** **±0.7**
**Insecurity**	
People are too dependent on technology to do things for them.	4.5 ± 1.0
Too much technology distracts people to a point that is harmful.	4.9 ± 1.0
Technology lowers the quality of relationships by reducing personal interaction.	4.7 ± 1.0
I do not feel confident interacting with someone that can only be reached online.	4.0 ± 1.1
Average score for insecurity	**4.5** **±0.7**
**Mean TRI Score**^**‡**^ **(telerehabilitation readiness)**	**3.3** **±0.4**

* *Cronbach's alpha >0.70*.

† *Responses ranged from 1 to 6 as follows: strongly agree = 6; somewhat agree = 5; neutral = 4; somewhat disagree = 3; strongly disagree = 2; or not sure = 1*.

‡ *TRI score per respondent was computed using the following formula: TRI = (Optimism + Innovativeness + [6-Insecurity] + [6-Discomfort]) / 4. Values in boldface represent average scores per dimension and overall TRI*.

The respondents had a mean telerehabilitation knowledge score of 2.1 ± 1.1 out of 5, interpreted as fair. Each item was correctly answered by <50% of the respondents, except for item number 2 in which almost 60% correctly identified the principles of informed consent (Table 3). On the other hand, <15% got the correct answer for the item regarding the minimum Internet bandwidth recommendation for a single healthcare provider practice.

**Table 3:** Tally of responses on the multiple-choice questions regarding telerehabilitation (*N* = 62).

**Telerehabilitation knowledge questions and choices**	**Responses, *n* (%)**
1. What is the recommended megapixel requirement of the web camera for optimal videoconferencing?	
a. 1–3	2 (3.2)
b. 3–5	15 (24.2)
c. 5–8^*^	**25 (40.3)**
d. >8	20 (32.3)
2. Which of the following is NOT included in the Principles of Informed Consent?	
a. Patient needs to be given the information.	4 (6.5)
b. Patient needs to understand the information.	0 (0.0)
c. Patient needs to make a choice.	21 (33.9)
d. Patient has to affix his/her signature above printed name to signify consent*.	**37 (59.7)**
3. Which simulation role is being described in the following statement? An individual with background in the health sciences; must be available at the originating site to present the patient, manage the cameras, and perform any hands-on activities; may sometimes provide information about the patient to the provider that the provider could not otherwise obtain.	
a. Clinic manager	21 (33.9)
b. Technical support	10 (16.1)
c. Telepresenter^*^	**28 (45.2)**
d. Receptionist/ scheduler	3 (4.8)
4. Which of the following is NOT included in the 3 main levels of risk-mitigation to ensure cyber-security?	
a. People who can access the system	6 (9.7)
b. Internet connectivity^*^	**29 (46.8)**
c. Technical components	14 (22.6)
d. The information itself	13 (21.0)
5. What is the recommended Internet bandwidth for a single healthcare provider practice?	
a.8 megabits per second (Mbps)	**25 (40.3)**
b. 6 Mbps	23 (37.1)
c. 2 Mbps*	9 (14.5)
d. None of the above	5 (8.1)

* *Correct answers. Values in boldface represent the most common response per item*.

The overall UTAUT score of the respondents was 4.5 ± 0.6 out of 5, interpreted as excellent telerehabilitation acceptance. The UTAUT constructs with the highest mean scores were: (1) behavioral intention (4.9 ± 0.9); (2) self-efficacy (4.7 ± 0.8); and (3) facilitating conditions (4.6 ± 0.8) (Table 4).

**Table 4:** Telerehabilitation acceptance of the respondents (*N* = 62) based on the modified Unified Theory of Acceptance and Use of Technology (UTAUT) questionnaire^*^.

**Items per UTAUT construct**	**Mean ±SD^**†**^**
**Performance expectancy:** the degree to which the respondent believes that telerehabilitation can help physiatrist and patient attain gains in healthcare	
I find telerehabilitation useful in my job.	4.9 ± 1.2
Using telerehabilitation enables me to accomplish tasks more quickly.	4.3 ± 1.4
Using telerehabilitation increases my productivity.	4.3 ± 1.3
If I use telerehabilitation, I will increase my chances of earning more in the future.	4.1 ± 1.4
Average score for performance expectancy	**4.4** **±1.2**
**Effort expectancy:** the degree to which the respondent believes that ease is associated with use of telerehabilitation	
My interaction with telerehabilitation could be clear and understandable.	4.4 ± 1.2
It could be easy for me to become skillful at using telerehabilitation.	4.4 ± 1.1
I find telerehabilitation easy to use.	4.2 ± 1.1
Learning to operate telerehabilitation is easy for me.	4.5 ± 1.1
Average score for effort expectancy	**4.4** **±1.0**
**Attitude:** the degree to which the respondent believes that using telerehabilitation is a good idea	
Using telerehabilitation is a good idea.	4.7 ± 1.0
Telerehabilitation makes work more interesting.	4.2 ± 1.2
Working with telerehabilitation is fun.	3.9 ± 1.2
I like working with telerehabilitation.	4.0 ± 1.3
Average score for attitude	**4.2** **±1.0**
**Social influence:** the degree to which the respondent perceives that his/her colleagues or institution believe/s he/she needs to use telerehabilitation	
People who influence my behavior think that I should use telerehabilitation.	4.2 ± 1.2
People who are important to me think that I should use telerehabilitation.	4.2 ± 1.2
Our department has been helpful in the use of telerehabilitation.	4.7 ± 1.1
In general, our department has supported the use of telerehabilitation.	5.1 ± 1.1
Average score for social influence	**4.5** **±0.9**
**Facilitating conditions:** the degree to which the respondent believes that an organization and infrastructure exist to support use of telerehabilitation	
I have the resources necessary to use telerehabilitation.	4.9 ± 1.1
I have the knowledge necessary to use telerehabilitation.	4.8 ± 1.0
Telerehabilitation is not compatible with other aspects of my work^‡^.	4.3 ± 1.2
A person or group inside or outside our department is available for assistance with telerehabilitation difficulties.	4.3 ± 1.3
Average score for facilitating conditions	**4.6** **±0.8**
**Self-efficacy:** the degree of the respondent's judgement to use telerehabilitation	
I could complete a job or task using telerehabilitation even if there was no one around to tell me what to do.	4.6 ± 1.1
I could complete a job or task using telerehabilitation if I could call someone for help if I got stuck^‡^.	4.7 ± 0.9
I could complete a job or task using telerehabilitation if I had a lot of time^‡^.	4.8 ± 1.0
I could complete a job or task using telerehabilitation if I had the built-in help facility for assistance^‡^.	4.7 ± 1.2
Average score for self-efficacy	**4.7** **±0.8**
**Anxiety:** the degree to which the respondent hesitates to use telerehabilitation	
I feel apprehensive about using telerehabilitation^‡^.	4.0 ± 1.1
It scares me to think that I could lose a lot of information using telerehabilitation^‡^.	4.7 ± 1.2
I hesitate to use telerehabilitation for fear of making mistakes I cannot correct^‡^.	4.2 ± 1.3
Telerehabilitation is somewhat intimidating to me^‡^.	3.6 ± 1.1
Average score for anxiety	**4.1** **±0.9**
**Behavioral intention:** the degree to which the respondent intends to use telerehabilitation	
I intend to use telerehabilitation in the next 6 months.	4.7 ± 1.2
I predict I would use telerehabilitation in the next 6 months.	5.1 ± 0.9
I plan to use telerehabilitation in the next 6 months.	4.9 ± 1.0
Average score for behavioral intention	**4.9** **±0.9**
**Mean UTAUT Score (Telerehabilitation Acceptance)**	**4.5** **±0.6**

* *Cronbach's alpha >0.70*.

† *Responses ranged from 1 to 6 as follows: strongly agree = 6; somewhat agree = 5; neutral = 4; somewhat disagree = 3; strongly disagree = 2; or not sure = 1*.

‡ *Scored reversely. Values in boldface represent average scores per construct and overall UTAUT*.

Based on linear regression, the following factors showed significant associations with telerehabilitation readiness: optimism, innovativeness, discomfort, and insecurity [F (9.612, 0.05) = 0.000, *p* < 0.05], with an R^2^ of 1.000. On the other hand, the rest of the variables (i.e., telerehabilitation knowledge, performance expectancy, effort expectancy, attitude, social influence, facilitating conditions, self-efficacy, anxiety, and behavioral intention) did not reach statistical significance (*p* > 0.05).

## Discussion

Our study showed that the Rehabilitation Medicine residents in the Philippines have good telerehabilitation readiness, fair telerehabilitation knowledge, and excellent telerehabilitation acceptance. Although the majority were classified as telerehabilitation skeptics (38.7%), combining telerehabilitation explorers (19.4%) and pioneers (19.4%), the two highest levels of technology adopters, comprised an almost equal percentage (38.8%). The factors that significantly influenced telerehabilitation readiness were the respondents' optimism, innovativeness, discomfort, and insecurity (*p* < 0.05), while telerehabilitation knowledge and UTAUT scores were not found to be statistically associated with readiness.

Our data was collected after more than 6 months into the pandemic. By then, the impact of the unprecedented COVID-19 crisis had become evident, particularly altering the way training, service, and research were conducted in the six Rehabilitation Medicine residency training programs, which are all located in Manila, the epicenter of the pandemic in the Philippines. It is not difficult to surmise that the residents, who are relatively young (~30 years of age), would have adapted to the sudden shift to virtual care and training, evidenced by their favorable telerehabilitation readiness and acceptance. However, their theoretical knowledge of telerehabilitation neither seemed adequate nor congruent with their readiness and acceptance.

It is established that many factors like knowledge, skills, attitude, and working environment contribute to the success of any new technology or its adoption (19). Telerehabilitation was neither widely taught nor practiced in the Philippines before the pandemic. As COVID-19 continues to cause a significant decline in the number of patients able and willing to access in-person rehabilitation, there is a need to strengthen the awareness, feasibility, and potential role of telerehabilitation among its stakeholders (5, 6). It is, therefore, important to establish a strong foundation of telerehabilitation principles among its target users, particularly the current and future clinicians who are considered the primary drivers of this emerging technology (10).

Traditionally, telehealth or telemedicine, let alone telerehabilitation, was not included in the curriculum of most, if not all, premedical, medical, and/or residency training programs in the Philippines (5, 20). In contrast, universities abroad mostly in developed countries like Australia, France, United Kingdom, and United States of America have had telehealth and/or telerehabilitation courses even before the pandemic (21, 22). Hence, an instructional design on telerehabilitation could possibly gain inspiration and guidance from existing formats from reputable institutions that have had wide experience in teaching telerehabilitation to students or professionals. Nonetheless, the curriculum design has to be adapted and contextualized according to the needs and resources in the local setting (5, 23, 24). Given that most, if not all, the current Rehabilitation Medicine residents have comparable levels of technological proficiency, the content of telerehabilitation training has to go beyond the basic and technical aspects and include ethical, legal, and socioeconomic principles applicable in their target area of practice (25).

Our study showed that almost half of the respondents did not have any telerehabilitation experience at the time of data collection. With the intermittent suspensions of outpatient rehabilitation services in Metro Manila and the prevailing apprehension of patients about in-person consultations and therapy sessions because of the unpredictable COVID-19 situation, the Rehabilitation Medicine residents encounter a significant decline of cases and, therefore, learning opportunities (6). Hence, telerehabilitation could be leveraged to augment their lack of clinical exposure (6). However, faculty and residents alike will need to adapt and relearn the conduct of routine physiatric history-taking and evaluation in the context of remote interaction. Acknowledging the inherent limitations of virtual physical examination, and ensuring benefits outweigh potential risks, the clinical principles of evaluating and managing various disabilities through telerehabilitation may have to be included in the curricular modifications of residency training in Rehabilitation Medicine.

Research interest in telerehabilitation has not been widely explored around the world until recently (11). Considered an emerging technology in the field of rehabilitation, telerehabilitation has yet to be given a globally agreed definition, scope, and standard practice among others. In the Philippines, as telerehabilitation continues to be a huge part of the daily service and training duties of residents in some institutions amid the enduring pandemic, it would be useful to develop a set of core competencies and evaluation methods to ensure that the standard practice of Rehabilitation Medicine is upheld in every virtual encounter. It would be a disservice to the patients if telerehabilitation were not conducted competently, ethically, and conscientiously.

Our study focused only on the current Rehabilitation Medicine residents. However, there are many other stakeholders that have to be evaluated as well, such as the patients and primary caregivers, consultant physiatrists, administrative staff, therapists, nurses, rural clinicians, and medical students. They could be potential targets of future research in order to determine their telerehabilitation perceptions. Exploratory qualitative studies about factors that could affect their telerehabilitation readiness, knowledge, and acceptance are recommended. Nonetheless to our knowledge, our study was the first in local and international literature to provide baseline data on the perceptions of physiatrists in-training regarding telerehabilitation. The design of our study and data may serve as benchmark for potential research on telerehabilitation education and training in the future.

## Conclusion

The Rehabilitation Medicine residents in the Philippines had mixed levels of telerehabilitation adoption, which ranged from being skeptics to pioneers and explorers. Although it seemed that despite their relatively low telerehabilitation knowledge and high telerehabilitation acceptance, their optimism and innovativeness seemed to be significant facilitators of telerehabilitation readiness. A call to action is warranted for incorporating telerehabilitation in the curriculum of Rehabilitation Medicine residency training to ensure quality of virtual care.

## Data Availability Statement

The raw data supporting the conclusions of this article will be made available by the authors, without undue reservation.

## Ethics Statement

This study was reviewed and approved by the University of the Philippines Manila-Research Ethics Board (UPM-REB). The participants provided their voluntary informed consent to participate in the study.

## Author Contributions

All authors listed have made a substantial, direct, and intellectual contribution to the work and approved it for publication.

## Funding

This study is a recipient of the Residents' Research Grant given by the Expanded Hospital Research Office of the Philippine General Hospital, University of the Philippines Manila.

## Conflict of Interest

The authors declare that the research was conducted in the absence of any commercial or financial relationships that could be construed as a potential conflict of interest.

## Publisher's Note

All claims expressed in this article are solely those of the authors and do not necessarily represent those of their affiliated organizations, or those of the publisher, the editors and the reviewers. Any product that may be evaluated in this article, or claim that may be made by its manufacturer, is not guaranteed or endorsed by the publisher.
